# The Genomic Ancestry of Individuals from Different Geographical Regions of Brazil Is More Uniform Than Expected

**DOI:** 10.1371/journal.pone.0017063

**Published:** 2011-02-16

**Authors:** Sérgio D. J. Pena, Giuliano Di Pietro, Mateus Fuchshuber-Moraes, Julia Pasqualini Genro, Mara H. Hutz, Fernanda de Souza Gomes Kehdy, Fabiana Kohlrausch, Luiz Alexandre Viana Magno, Raquel Carvalho Montenegro, Manoel Odorico Moraes, Maria Elisabete Amaral de Moraes, Milene Raiol de Moraes, Élida B. Ojopi, Jamila A. Perini, Clarice Racciopi, Ândrea Kely Campos Ribeiro-dos-Santos, Fabrício Rios-Santos, Marco A. Romano-Silva, Vinicius A. Sortica, Guilherme Suarez-Kurtz

**Affiliations:** 1 Departamento de Bioquímica e Imunologia, Universidade Federal de Minas Gerais, Belo Horizonte, Brazil; 2 Departamento de Ciências da Saúde, Universidade Estadual de Santa Cruz, Ilhéus, Brazil; 3 Coordenação de Pesquisa/Divisão de Farmacologia, Instituto Nacional do Câncer, Rio de Janeiro, Brazil; 4 Departamento de Genética, Universidade Federal do Rio Grande do Sul, Porto Alegre, Brazil; 5 Instituto Nacional de Ciência e Tecnologia de Medicina Molecular (INCT-MM), Laboratório de Neurociência, Faculdade de Medicina, Universidade Federal de Minas Gerais, Belo Horizonte, Brazil; 6 Unidade de Farmacologia Clínica, Departamento de Fisiologia e Farmacologia, Faculdade de Medicina da Universidade Federal do Ceará, Fortaleza, Brazil; 7 Laboratório de Genética Humana e Médica, Universidade Federal do Pará, Guamá, Brazil; 8 Laboratório de Neurociências, Instituto de Psiquiatria, Faculdade de Medicina, Universidade de São Paulo, São Paulo, Brazil; University of Utah, United States of America

## Abstract

Based on pre-DNA racial/color methodology, clinical and pharmacological trials have traditionally considered the different geographical regions of Brazil as being very heterogeneous. We wished to ascertain how such diversity of regional color categories correlated with ancestry. Using a panel of 40 validated ancestry-informative insertion-deletion DNA polymorphisms we estimated individually the European, African and Amerindian ancestry components of 934 self-categorized White, Brown or Black Brazilians from the four most populous regions of the Country. We unraveled great ancestral diversity between and within the different regions. Especially, color categories in the northern part of Brazil diverged significantly in their ancestry proportions from their counterparts in the southern part of the Country, indicating that diverse regional semantics were being used in the self-classification as White, Brown or Black. To circumvent these regional subjective differences in color perception, we estimated the general ancestry proportions of each of the four regions in a form independent of color considerations. For that, we multiplied the proportions of a given ancestry in a given color category by the official census information about the proportion of that color category in the specific region, to arrive at a “total ancestry” estimate. Once such a calculation was performed, there emerged a much higher level of uniformity than previously expected. In all regions studied, the European ancestry was predominant, with proportions ranging from 60.6% in the Northeast to 77.7% in the South. We propose that the immigration of six million Europeans to Brazil in the 19^th^ and 20^th^ centuries - a phenomenon described and intended as the “whitening of Brazil” - is in large part responsible for dissipating previous ancestry dissimilarities that reflected region-specific population histories. These findings, of both clinical and sociological importance for Brazil, should also be relevant to other countries with ancestrally admixed populations.

## Introduction

Continental populations of the world vary considerably in their predisposition to diseases and in the allele frequencies of important pharmacogenetic loci, probably as a result of genetic drift, but also because of adaptation to local selective factors such as climate and available nutrients. In many countries, skin color has traditionally been used in clinical and pharmacological studies as a phenotypic proxy for geographical ancestry. Brazil is no exception.

The Brazilian population was formed by extensive admixture from three different ancestral roots: Amerindians, Europeans and Africans. This resulted in a great variability of skin pigmentation, with no discontinuities between Black and White. For instance, in a single small fishing village in Brazil, Harris and Kotak [1] identified dozens of designations for varied shades of skin pigmentation.

However, the Instituto Brasileiro de Geografia e Estatística (IBGE), which is responsible for the official census of Brazil, has employed only few pre-established color categories, which are based on self-classification. Since 1991 they number five: White (“branca”), Brown (“parda”), Black (“preta”), Yellow (amarela) and Indigenous (“indígena”). Brown (“pardo”) emerged as a synthesis of a variety of classifications, such as “caboclo”, “mulato”, “moreno”, “cafuzo”, and other denominations that express the admixed character of the Brazilian population [2].

In general, there is academic support for the IBGE classification system, which is the only source of information on color categories at a national level [3], [4]. It reflects the fact that in Brazil social “racial” categorization depends not on ancestry, but on the physical appearance of the individual [5].

In 2008 IBGE ascertained a population of *circa* 190 million Brazilians who, based on self-classification, could be segregated into the following proportions for color: 48.4% White, 43.8% Brown, 6.8% Black, 0.6% Yellow, 0.3% Indigenous and 0.1% with no declaration (http://www.sidra.ibge.gov.br/bda/tabela/listabl.asp?z=t&c=262). The first three of these categories (White, Brown and Black) encompass 99.1% of the Brazilian population and will be the focus of this study. It is important to realize that in Brazil, color (in Portuguese, *cor*) denotes the Brazilian equivalent of the English term race (*raça*) and is based on a complex subjective phenotypic evaluation that takes into account, not only skin pigmentation, but also hair pigmentation and type, eye melanization and facial features such as nose and lip shape [6].

With an area of 8,511,960 Km^2^, Brazil has a territory of continental size (the fifth largest in the world) and different regions have diverse population histories. For instance, the North had a large influence of the Amerindian root, the Northeast had a history of strong African presence due to slavery and the South was mostly settled by European immigrants. These different compositions were quite evident in our studies of mtDNA haplotypes of White Brazilians [7], [8].

When we look into the Brazilian census data on the proportion of each color category according to region, we indeed can see noticeable differences (Table 1). In the North and Northeast there is a strong predominance of Brown individuals (64.0% and 58.0%, respectively) while in the Southeast and South, White Brazilians constitute the largest category (62.4% and 83.6%, respectively). The Center-West, the least populous region, includes the heterogeneous Federal District (i.e. Brasilia) and displays more even proportions of White and Brown individuals (49.7% and 43.7%, respectively). Such high level of regional structure is peculiar, especially when we consider that our previous work has shown only a feeble relationship between color and ancestry in Brazilians [8]–[10].

**Table 1:** 2008 IBGE data for the regions and states sampled in this study.

		Population	White	Brown	Black
		(X 10^3^)			
Brazil		189,953	92,003	83,196	12,987
			(48.43%)	(43.80%)	(6.84%)
Region	State				
North	Pará	7,367	1,530	5,374	398
		(3.88%)	(20.77%)	(72.95%)	(5.40%)
	Ceará	8,472	2,800	5,370	257
Northeast		(4.46%)	(33.05%)	(6339	(303
	Bahia	14,560	2,999	9,149	2,328
		(7.67%)	(20.60%)	(62.84%)	(15.99%)
Southeast	Rio de Janeiro	16,203	8,509	5,302	2,328
		(8.53%)	(52.51%)	(32.72%)	(14.37%)
	Santa Catarina	6,091	5,297	608	160
South		(3.21%)	(86.96%)	(9.98%)	(2.63%)
	Rio Grande	10,856	8,776	1,495	529
	do Sul	(5.72%)	(80.84%)	(13.77%)	(4.87%)

The first column shows the total population of Brazil and the population of each state expressed in absolute values and percentage of the total for the whole Country. The columns for the color categories contain data also expressed in absolute numbers and percentages self-categorized in that region (in parentheses). The percentages for Whites, Blacks and Browns do not add to 100% because each State has individuals who belong to color categories that are distinct from the ones shown. Data obtained from http://www.sidra.ibge.gov.br/bda/tabela/listabl.asp?z=t&c=262.

We have already shown that a set of 40 short insertion-deletion (indel) polymorphisms was sufficient for an adequate characterization of human population structure at the global level [11]. We furthermore demonstrated the resolution power of these markers in discriminating among Europeans, Africans and Amerindians by plotting in a triangular graph our results with the samples of Europeans, Africans and Amerindians of the HGDP-CEPH Diversity Panel [12]. Three totally divergent clusters that correspond to the European, African and Amerindian populations were obtained without any overlap — each group clustered in one of the vertices of the triangular plot [8].

In the present study we used these loci to estimate the Amerindian, European and African genomic ancestry of 934 Brazilians from the four most populous geographical regions of the Country, self-categorized as White, Brown and Black.

## Results

### Estimates of the trihybrid ancestry of Brazilians from different regions

**Figure 1:**
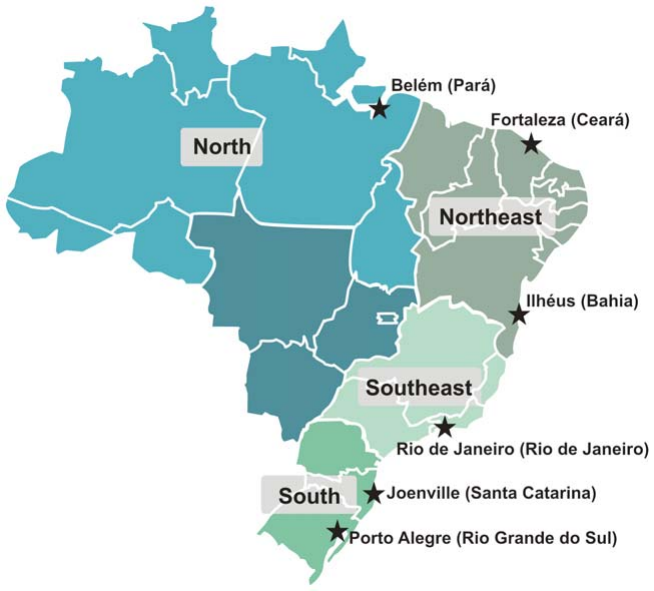
Map of Brazil showing the five geographical regions of the country. The regions with a square label were analyzed in this work. The cities and respective states where the samples were collected are shown with a star.

**Figure 2:**
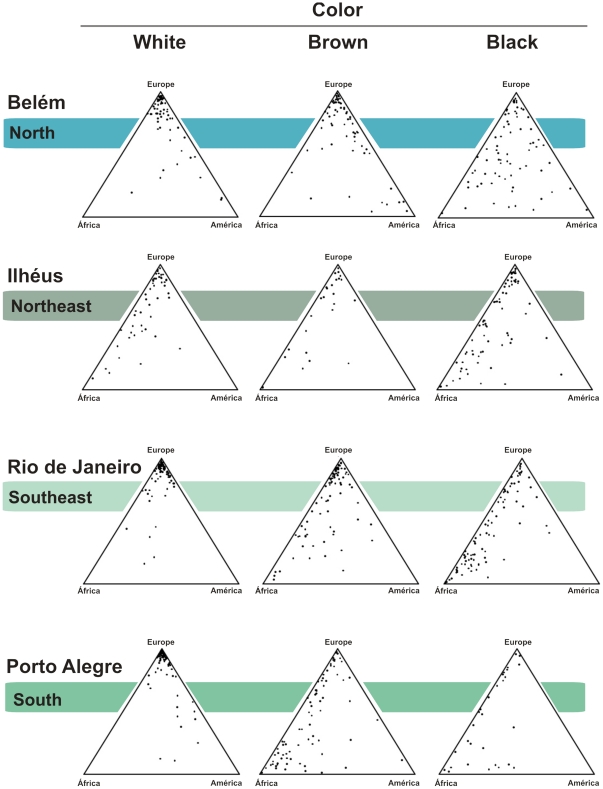
Triangular plots of the genomic proportions of African, European and Amerindian ancestry in three self-reported color groups of 934 Brazilian individuals from four different regions of the country, self-categorized White, Brown and Black individuals. Each point represents a separate individual and the ancestral proportions can be determined by dropping a line parallel to each of the three axes. The graphs were drawn using the Tri-Plot program [42].

In the present work we established the genotype of 934 self-classified White, Brown or Black Brazilians at 40 autosomal short insertion-deletion polymorphisms (indels) dispersed in the human genome. The allele frequencies at these loci are shown in Table S1. We then used the genotypes and the *Structure* program to estimate, at an individual level, the European, African and Amerindian components of ancestry for these individuals from states in four geographical regions of Brazil (Fig. 1). All the individual estimates are shown in triangular plots in Fig. 2. We then calculated the mean and standard error of individual estimates to arrive at a summarizing figure for each group (Table 2).

**Table 2:** Mean and standard error for the estimated Amerindian, European and African ancestries of 934 Brazilian individuals from four regions of the country, self-categorized as having White, Brown and Black color.

Region	Ancestral Roots	Color category						Color-independent “Total Ancestry”
		White		Brown		Black		
		Mean	s.e.	Mean	s.e.	Mean	s.e.	
North	European	0.782	0.026	0.686	0.034	0.524	0.031	0.697
(Pará)	African	0.077	0.011	0.106	0.016	0.275	0.023	0.109
	Amerindian	0.141	0.022	0.209	0.030	0.201	0.026	0.194
Northeast	European	0.668	0.037	0.603	0.060	0.539	0.034	0.606
(Bahia)	African	0.244	0.033	0.308	0.057	0.359	0.014	0.303
	Amerindian	0.088	0.012	0.089	0.020	0.101	0.031	0.091
Northeast	European	0.758	0.032	0.728	0.029	N.S.	N.S.	
(Ceará)	African	0.133	0.017	0.144	0.021	N.S.	N.S.	
	Amerindian	0.109	0.021	0.128	0.015	N.S.	N.S.	
Southeast	European	0.861	0.016	0.675	0.028	0.427	0.032	0.737
(Rio de Janeiro)	African	0.074	0.011	0.238	0.025	0.495	0.032	0.189
	Amerindian	0.065	0.007	0.087	0.012	0.079	0.009	0.074
South	European	0.855	0.021	0.442	0.037	0.431	0.062	0.777
(Rio Grande do Sul)	African	0.053	0.019	0.444	0.035	0.459	0.052	0.127
	Amerindian	0.093	0.006	0.114	0.016	0.110	0.026	0.096
South	European	N.S.	N.S.	N.S.	N.S.	0.293	0.031	
(Santa Catarina)	African	N.S.	N.S.	N.S.	N.S.	0.596	0.030	
	Amerindian	N.S.	N.S.	N.S.	N.S.	0.111	0.012	

N.S.  =  Not studied.

The most evident diversity in the ancestral Amerindian, European and African proportions of the different color categories, both between and within the different regions of Brazil, was seen in individuals self-assessed as Brown. For instance, in the North (Belém, PA) self-classified Brown individuals had, on the average, 68.6% European ancestry, followed by 20.9% Amerindian ancestry and 10.6% African ancestry, while in the South they had, on the average, 44.2% European, 11.4% Amerindian and 44.4% African ancestries.

To estimate the significance of the pairwise differences observed between the samples of individuals self-classified as Brown in diverse regions, we used a specially designed Monte Carlo randomization test of the distance D between the means, described in detail in the Material and Methods section. In Table S2 the observed distances are in the cells above the diagonal and the probability of obtaining the observed distances by chance is shown in the cells below the diagonal.

Since we have six comparisons, we need to control for type I error. Applying the Bonferroni correction [13], [14] the alpha value for significance was reduced to 0.008. Even then, four of the six contrasts are significant, i.e. between North (Pará) and Southeast (Rio de Janeiro); between North (Pará) and South (Rio Grande do Sul); between the Northeast (Ceará) and the South (Rio Grande do Sul) and between the Southeast (Rio de Janeiro) and the South (Rio Grande do Sul). This reinforces the idea that the semantic criteria for color self-categorization are heterogeneous in the different Brazilian regions.

### Estimates of the “total ancestry” of different regions of Brazil

Since both the census proportions of each color category and the trihybrid ancestry of Brazilians vary according to region, we decided to merge the two sets of data and estimate what we have called the “total ancestry” of a given region. This has the advantage of circumventing the different regional semantics of what it means “to be” White, Brown or Black. To calculate the total ancestry we simply multiply the proportions of a given ancestry in a given color category by the census proportion of that color category in the specific region to arrive at an ancestry estimation regardless of color.

In order to show how the calculation of the “total ancestry” was done, let us take the example of European ancestry in the North region (state of Pará) using the data from Table 2. In that state, White, Brown and Black individuals have average European ancestry of 0.782, 0.686 and. 0.524 respectively. Since for the state of Pará the census shows the relative proportions of the same three colors above as 0.210, 0.736 and 0.055, the weighted European ancestry, which is now independent of color, will be (0.782×0.210) + (0.686×0.736) + (0.524×0.055)  = 0.697.

The “total ancestry” estimates thus calculated for all regions are shown in the rightmost column of Table 2. The calculation could not be performed for two of the samples, Ceará and Santa Catarina, because they lacked data on one or more color categories.

The results obtained showed that there is in fact a smaller level of variability between the different regions than had been observed in the census data of color categories or in the ancestry proportions of the different color classes (Fig. 1). In all regions studied the European ancestry surfaced as uniformly preponderant, with proportions of 69.7%, 60.6%, 73.7% and 77.7%, respectively (Table2). This suggests that the populations of different regions of Brazil are ancestrally more similar than previously realized.

## Discussion

### The trihybrid ancestry of Brazilians from different regions

We here present results of the molecular estimation of the European, African, and Amerindian ancestry in 934 individuals belonging to different color categories and originated from four regions of Brazil (Figure 1). The picture that emerges from the data is of great heterogeneity both within and between color categories and geographical regions (Figure 2 and Table 2). A noteworthy observation is that there is considerable admixture, which can be appreciated both at the individual and the group level. Individually, triangular plots show great variation in ancestry levels for all color categories and in all regions (Fig. 1). At a group level, for instance, we can observe that the African ancestry of Black individuals is below 50% in all samples tested, with the exception of the state of Santa Catarina, in the South.

In a previous publication [9], with different samples, a weak relationship had already perceived between color and geographic genomic ancestry in Brazilians at an individual level, as we also observed in the present study. If we consider some peculiarities of Brazilian history and social structure, we can understand why indeed this should be so. Africans characteristically have black skin associated with other iconic individual components of color (black curly hair, black eyes, broad nose and thicker lips) and Europeans have white skin associated iconic individual components (light straight hair, light-colored eyes and thin nose and lips), all genetically determined by a relatively small number of genes that were evolutionarily selected by the geographical environment, especially the prevailing levels of sun UV exposure [15]. Thus, if we have a social race identification system based primarily on phenotype, such as occurs in Brazil, we classify individuals on the basis only of the presence of certain alleles at a relatively small number of genes that have impact on color, while ignoring the rest of the genome (where the 40 indels that we used to estimate ancestry are located). Now, if we have a population that is produced by extensive admixture of Europeans and Africans, as we know happened in Brazil, the association between ancestry and color should dissipate with time, which is exactly what we have seen.

### The heterogeneity in the ancestry estimates of different color categories in different regions

Another important observation is the considerable variability in the ancestry of color categories in different regions, most manifest in Brown and Black individuals. For instance, self-classified Brown individuals from the North had on the average 68.6% European ancestry, while in the South they had on the average 44.4% African ancestry. Also, for individuals self-classified as Black we can see considerable, but highly discrepant levels of European ancestry varying from 29.3% in Santa Catarina to 53.9% in Bahia. The most uniform category was that of individuals self-classified as White who consistently had a predominant European ancestry, varying from 66.8% in Bahia (BA) to 85.5% in Rio Grande do Sul and 86.1% in Rio de Janeiro.

It is noteworthy that such different regional subjective differences in color perception unraveled by our ancestry analysis appear to run counter to expectations based on pre-genomic racial/color methodology. For instance, Osorio [4] discusses a hypothetical case of twins with identical ancestry proportions and with phenotypes in the White-Brown frontier and who have been raised separately, one in Bahia and the other in South Brazil. He then states that it would be expected that the one in Bahia should be considered White while the other, in the South, would be considered Brown or Black. But what our data shows is the opposite: Browns and Black individuals in Bahia have in fact less African ancestry than their counterparts in the South.

One possible explanation for this might be the effect of darker pigmentation by sun exposure. Jablonski and Chaplin [16] have shown that skin reflectance is strongly correlated with absolute latitude and UV radiation levels. This is due in large part to environmental factors, i.e., UV exposure [15]. Brazil occupies a subtropical position and the UV exposure in the North and Northeast is higher than in the Southeast and considerably higher than in the South. Thus, it might be necessary to have a higher level of African Ancestry present to have a Brown or Black skin color in the South, with lesser sun exposure, than in the northern regions of Brazil. A second, additive, possibility is that there are different cultural semantic criteria for color classification in diverse regions.

Independent of the reason, it is evident that ancestrally people who are White, Brown or Black in the northern part of Brazil are different from their counterparts in the southern part of the Country. This shows that, as has been pointed out before [9], the relationship between color and geographical ancestry is tenuous and one cannot use interchangeably terms such as White, Caucasian and European in one hand, and Black, Negro or African in the other, as is often done in daily discourse, in political rhetoric and in the medical and scientific literature.

### Total ancestries

**Figure 3:**
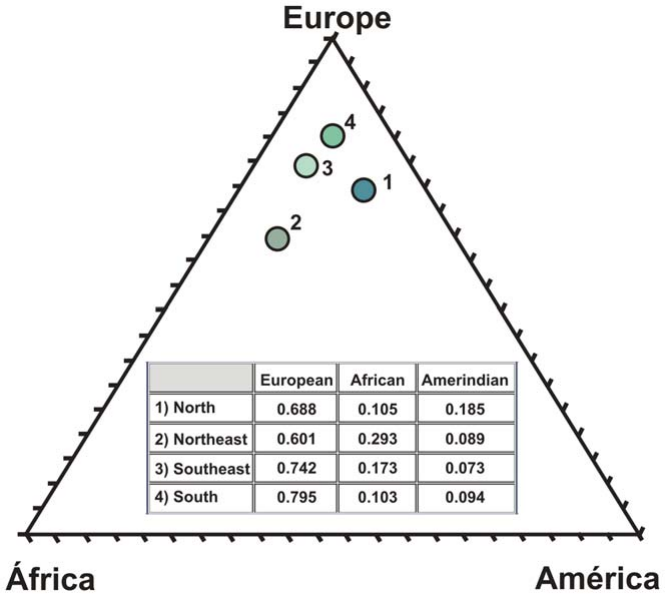
Triangular plot and table of the genomic proportions of African, European and Amerindian ancestry in four different regions of Brazil, independent of color category. Each point represents a separate region, as follows (1) North (Pará), (2) Northeast (Bahia), (3) Southeast (Rio de Janeiro) and (4) South (Rio Grande do Sul). The graph was drawn using the Tri-Plot program [42].

To eschew the use of color categories we decided to try to estimate the general ancestry proportions of the different regional samples independent of color categories. To do that, we multiplied the proportions of a given ancestry in a given color category by the census proportion of that color category in the specific region, to arrive at ancestry estimation independent of color. Once such a correction was performed on the basis of the relative proportion of Amerindian, European and African ancestries, there emerged a higher level of uniformity than expected. In all regions studied the European ancestry was predominant, with proportions being ranging from 60.6% in the Northeast to 77.7% in the South (Figure 3). The African proportion was highest in the Northeast (30.3%), followed in decreasing order by the Southeast (18.9%), South (12.7%), and North (10.9%). On the other hand, the Amerindian proportion was highest in the North (19.4%), while relatively uniform in the other three other regions.

This is novel genetic information about the Brazilian people that needs to be placed on a historical and phylogeographical context. First, we will compare them with our previous observations with uniparental genetic markers in Brazilians.

We earlier examined DNA polymorphisms in the non-recombining portion of the Y-chromosome and in the hypervariable region of mitochondrial DNA (mtDNA) in the four main regions of the Country (the same four regions analyzed in the present paper, although with samplings from different states). The vast majority of Y-chromosomes, independent of the region, proved to be of European origin [17], [18]. Studies of mtDNA revealed a different reality: considering Brazil as a whole, 33%, 39% and 28% of matrilineages were of Amerindian, European and African origin, respectively [7]. Significantly, the frequency of mtDNA ancestries varied a lot in different regions: most matrilineal lineages in the Amazonian region had Amerindian origin (54%), while African ancestry was preponderant in the Northeast (44%) and European haplogroups were prevalent in the South (66%). These data have since been amply confirmed by other studies [8]. Together, they configured a picture of strong directional mating between European males and Amerindian and African females, which agrees perfectly with the known history of the peopling of Brazil since 1500 [8].

The proportions of Amerindian and African maternal ancestry were higher in the previous investigation using mtDNA than in the regional total ancestry averages calculated in the present study using biparental markers. However, it is interesting to note that both studies agree in that the highest level of Amerindian ancestry could be found in the North region (54% for mtDNA; 19.4% in the present study) and the highest level of African ancestry belonged to the Northeast region (44% for mtDNA; 30.3% in the present study), exactly as expected from known historical and anthropological studies of Brazilians [19].

### A unifying proposal

As mentioned previously, Brazil is the home of genetically heterogeneous people, the product of five centuries of admixture between Amerindians, Europeans and Africans. However, such admixture has occurred in a sexually asymmetric fashion, as a result of the colonization model employed by the Portuguese. Indeed, we know that few women came from Portugal to Brazil in the period from the arrival of the Europeans in 1500 until 1808, when the Portuguese Court fled the Napoleonic invasion of the Iberian Peninsula and relocated to Rio de Janeiro [20]. That means that the Brazilian population was primarily formed by male Portuguese and female native Amerindian and enslaved Africans.

Initially, the whole population was composed by the indigenous Amerindians. Little is known about their number when the Portuguese arrived in 1500 [21], although a figure often cited is that of 2.5 million individuals [21]. From 1500 to 1808, it is estimated that about 500,000 Portuguese colonizers, almost exclusively men, came to Brazil [20], admixing extensively with the Amerindian women. Thus, we can expect that the first generation of Brazilians genomically had 50% Amerindian and European ancestry, but 100% Amerindian mtDNA. Further generations of admixture with the Portuguese lead to progressive “europeanization” of genomic ancestry, while maintaining an elevated proportion of Amerindian mtDNA.

The slave traffic started in the middle of the 16^th^ century, extending until 1850 and resulting in the forced relocation of an estimated 4 million Africans to Brazil [21]. These three centuries were a period of intense interbreeding between European males and Amerindian and African women, which led to introgression of genomic African ancestry into Brazilians and also of African mtDNA, since the African contribution was primarily from females [7].

Let us take, as a generic example, the mating of a white European male with a Black African slave woman in Brazil. Because of the Brazilian social race identification system based primarily on phenotype, the children with dark skin pigmentation and other African iconic individual components of color would be considered Black, while those with light colored skin and other European iconic individual components of color would be considered White, even though they would have exactly the same proportion of African and European alleles [9]. Since in Brazil there also occurs assortative mating by color (as has indeed been revealed by demographic studies) [22], [23], in the hypothetical subsequent generation, the light-skinned individuals would tend to marry other Whites and conversely the dark-skinned individuals would marry Blacks. The long-term tendency would then be for this pattern to produce two distinct color groups, White and Black, which would, nonetheless, both have simultaneously a significant level of European and African ancestry.

It is relevant to notice that 1.72 million slaves (42.9% of the total) arrived in Brazil during the first half of the 19^th^ century, a time by which the number of Amerindians in Brazil had dwindled due to strife and/or European-borne disease. Most likely, the main contribution of Amerindians to the formation of the Brazilian people occurred in the first 2 or at most 3 centuries of its colonization, no longer being of high importance in the early 19^th^ century, when larger and larger portions of Brazilians moved from rural areas to the cities. Since Africans (up until 1850) and Europeans (up until the 20^th^ century) continued to arrive to Brazil and to participate in the gene pool, the Amerindian ancestry component was diluted across color-lines to the levels that we observe presently, but without losing its mtDNA representativity because of the sexual asymmetry of the relationships. The resulting highly admixed Brazilian population can be assessed by the proportions of the color categories in first Brazilian census in 1872, which was 19.7% Black, 42.2% Brown and 38.1% White.

In 1850, the forced arrival of Africans stopped due to prohibition of the slave trade. At the same time the Government started a campaign to stimulate the immigration of Europeans to Brazil. This process, which has been denominated the “Whitening of Brazil” had complex economic and sociological causes, and was tinged with racist ideology [24]–[27]. In the approximately one hundred year period 1872–1975, Brazil received 5,435,735 million immigrants from Europe and the Middle East [21]. These were, in decreasing percentages, 34% Italians, 29% Portuguese, 14% Spanish, 5% Japanese, 4% Germans, 2% Lebanese and Syrians and 12% others [21].

This huge demographic event is probably responsible for the noteworthy dissipation of previously established regional differences in ancestries, as the European component of ancestry became uniformly preponderant, with similar proportions of 69.7%, 60.6%, 73.7% and 77.7% in the North, Northeast, Southeast and South, respectively.

How to explain why no similar wash-out occurred in respect to the matrilineal ancestry? We believe that the regional disparities in mtDNA ancestry were maintained because, once again, in the immigratory wave of Europeans there was a significant excess of males. When they admixed with the Brazilian women there was rapid europeanization of the genomic ancestry, but preservation of the established matrilineal pattern. There is demographic information to corroborate this possibility. First, of 1,222,282 immigrants from all origins that arrived in the Port of Santos in the period 1908–1936 the sex ratio (males/females) was 1.76 [28]. Second. the two most abundant immigrants, Portuguese and Italians, had sex rations of 2.12 and 1.83, respectively. census data of 1910 showed concordant results: there were 1,138,582 foreigners in Brazil, with a male/female ratio of 1.74, while there were 22,275,595 Brazilians with an even sex ratio of 1.02 [29].

### Clinical Implications

Understanding the heterogeneity and admixture of Brazilians within and between geographical regions has important clinical implications for the design and interpretation of clinical trials, the practice of clinical genetics and genomic medicine, the implementation of pharmacogenetic knowledge in drug prescription, and the extrapolation of data from other, more homogeneous populations.

Let us take the case of VKORC1, a key enzyme of the vitamin K cycle that is a molecular target of the coumarin anticoagulant warfarin. Polymorphisms of the *VKORC1* gene vary markedly in frequency worldwide, and this diversity is a major determinant of the individual dose requirement and clinical response to warfarin and other anticoagulants in several populations [30]. For example, the higher frequency of the warfarin-sensitive *VKORC1* 1173T variant allele in Japanese (0.89) compared to Caucasians (0.42) explained why the median warfarin dose is significantly higher in Caucasian than Japanese patients [31]. Accordingly, Limdi et al [32] have recently shown that differences in the warfarin dose requirements explained by *VKORC1* across several populations worldwide are largely accounted by the minor allele frequency of the *VKORC1* 1173C>T and 3673G>A (alternatively known as -1639G>A) SNPs.

We genotyped the *VKORC1* polymorphisms 3673G>A (rs99232315), 808T>G (rs2884737), 6853G>C (rs8050894) and 9041G>A (rs7294) in the same individuals analyzed in the present article [33]. We then inferred the statistical association between the distribution of the *VKORC1* haplotypes among Brazilians and self-reported color, geographical region and genetic ancestry by fitting multinomial log linear models via neural networks. The frequency distribution of the *VKORC1* haplotypes among Brazilians varied across geographical regions and self-reported color categories. Notably, the frequency of the warfarin sensitive *VKORC1* 3673A allele and the distribution of *VKORC1* haplotypes varied continuously as the individual proportion of European ancestry increased in the entire cohort, independently of race/color categorization and geographical origin. We concluded that warfarin dosing algorithms that include ‘race’ terms defined for other populations are clearly not applicable to the heterogeneous and extensively admixed Brazilian population.

Another example was provided by the *CYP3A5* gene, which encodes the enzyme CYP3A5, responsible for the inactivation of several clinically useful drugs, such as the immunosuppressants tacrolimus and cyclosporine. The frequency of the variant CYP3A5*3 allele (rs776746), which encodes a non-functional CYP3A5 isoform, is less than 10% among sub-Saharan Africans but exceeds 90% among Europeans. However, we have shown that the frequency of CYP3A5*3 in healthy Brazilians living in Rio de Janeiro, self-identified as White or Black according to the “color/race” categorization of the Brazilian census, was 78% and 32%, respectively, with enormous individual variability within the groups [34]. Thus, the CYP3A5*3 allele was three times more frequent among self-identified black Brazilians (32%) than black Africans (<10%) and was considerably less common among self-identified white Brazilians (78%) than Europeans (>95%). The African-European admixture of Brazilians provides an explanation for these discrepancies, since irrespective of “color/race” self-identification, most Brazilians share European and African genetic ancestries, and many have also a significant proportion of Amerindian ancestry.

These results show that the heterogeneity of our population cannot be adequately represented by arbitrary “race/color” categories. In a pharmacogenetic context, this implies that each person must be treated as an individual rather than as an “exemplar of a color group” [35].

Based on traditional demographic racial/color methodology, clinical and pharmacological trials in Brazil have usually considered the different regions of the Country as very heterogeneous. Our results show that when viewed under the light of molecular population genetics these classical paradigms are inadequate, since the genomic ancestry of individuals from different geographical regions of Brazil is more uniform than expected.

Our results have considerable sociological relevance for Brazil, because the race question presently figures prominently in Brazilian political life [36]. Among the actions of the State in the sphere of race relations are initiatives aimed at strengthening racial identity, especially “Black identity” encompassing the sum of those self-categorized as Brown or Black in the censuses and government surveys. The argument that non-Whites constitute more than half of the population of the country has been routinely used in arguing for the introduction of public policies favoring the no-White population, especially in the areas of education (racial quotas for entrance to the universities), the labor market, access to land, and so on [36]. Nevertheless, our data presented here do not support such contention, since they show that, for instance, non-White individuals in the North, Northeast and Southeast have predominantly European ancestry and differing proportions of African and Amerindian ancestry.

The relevance of our work also extrapolates the Brazilian borders. Because of its heterogeneous Amerindian, European and African ancestral roots, Brazil has been an important model for the population genetics and pharmacogenetics of admixed populations. Our article demonstrates how critical it is to use genomic tools to reevaluate and modernize previous regional population models established using conventional demographic, anthropological and sociological studies. The same should also be applied to other countries that contain ancestrally admixed populations.

## Materials and Methods

### Ethics statement

The Research Ethics Committee of the Instituto Nacional do Câncer (INCA) approved in July 15, 2005 the protocol of the study “Characterization of polymorphisms of pharmacogenetic interest and correlation with genetics ancestry” as well as the written Informed Consent form. In August 11, 2008 the Research Ethics Committee of the Instituto Nacional do Câncer (INCA) approved the enlargement of the study and carried forward the approval of the written consent Informed Consent form. The samples were anonymized after collection.

### Populations Studied

We studied 934 unrelated Brazilians from different geographical regions of Brazil (Fig. 1), as described in detail below. Except when noted, the color assignation was obtained by self-assessment in answer to the closed question “What is your color/race?”, as done in the Brazilian census by the Instituto Brasileiro de Geografia e Estatística (IBGE). All subjects of this study described themselves as White, Brown or Black (in Portuguese, respectively, “Branco”, “Pardo” and “Preto”). These three color categories encompass 99.1% of the Brazilian population. No subjects were self-classified as Indian (“Indígena”), Yellow (“Amarelo”) or did not declare a color (“Sem declaração”).

The North region was represented by 203 unrelated, healthy individuals (92 men, 111 women) from the Amazonian state of Pará (PA - Fig. 1). The individuals were ascertained in the Municipal Health Clinic in the Sacramenta area of the city of Belém, Pará and self-identified as White (n = 66; 33%), Brown (n = 65; 32%) or Black (n = 72; 35%). They were randomly chosen within each color category.

Two different samples were collected in the Northeast region: (i) 82 individuals were ascertained from healthy students and work personnel at the University of Ceará, in Fortaleza, Ceará (CE - Fig. 1), randomly chosen within the self-classified categories of White (n = 31) or Brown (n = 51) and (ii) 147 healthy individuals were ascertained from the Blood Bank in the city of Ilhéus, Bahia (BA - Fig. 1), randomly chosen within those self-classified as White (n = 48), Brown (n = 26) or Black (n = 73), as described elsewhere [37].

The Southeast sample was made up of 264 unrelated, healthy individuals (162 men, 102 women) from the state of Rio de Janeiro (RJ - Fig. 1), all collected from blood donors, personnel and research students at the Instituto Nacional do Cancer (INCA). The enrolled individuals were self-identified as White (n = 88; 33%), Brown (n = 88; 33%) or Black (n = 88; 33%). They were randomly chosen within each color category. Some of these subjects were analyzed in previous publications [34], [38]. The genotype results were very similar with those from a different study with another populations of the city of Rio de Janeiro [38].

Two different samples were obtained in the South region: (i) 189 individuals ascertained from blood donors in Porto Alegre, Rio Grande do Sul (RS - Fig. 1), were not self-identified, but rather had their color assigned by the health professionals who collected their samples, as White (n = 82), Brown (n = 78) or Black (n = 29). They were randomly chosen within each color category. Studies performed in Southern Brazil with large samples have shown that for White interviewers (which was the case in our study) there was no statistical difference between self-classification and interviewer-classification [39] and (ii) 49 self-assessed Black individuals from a Community Center in the city of Joinville, Santa Catarina (SC - Fig. 1).

### DNA analysis

DNA from each individual was independently typed for the following 40-biallelic short insertion/deletion polymorphisms (indels): MID-1 (rs3917), MID-15 (rs4181), MID-17 (rs4183), MID-51 (rs16343), MID-89 (rs16381), MID-107 (rs16394), MID-131 (rs16415), MID-132 (rs16416), MID-150 (rs16430), MID-159 (rs16438), MID-170 (rs16448), MID-258 (rs16695), MID-278 (rs16715), MID-420 (rs140709), MID-444 (rs140733), MID-468 (rs140757), MID-470 (rs140759), MID-663 (rs1305047), MID-788 (rs1610874), MID-857 (rs1610942), MID-914 (rs1610997), MID-918 (rs1611001), MID-1002 (rs1611084), MID-1092 (rs2067180), MID-1100 (rs2067188), MID-1129 (rs2067217), MID-1291 (rs2067373), MID-1352 (rs2307548), MID-1428 (rs2307624), MID-1537 (rs2307733), MID-1549 (rs2307745), MID-1586 (rs2307782), MID-1642 (rs2307838), MID-1654 (rs2307850), MID-1759 (rs2307955), MID-1763 (rs2307959), MID-1847 (rs2308043), MID-1861 (rs2308057), MID-1943 (rs2308135), MID-1952 (rs2308144). In this list, The MID number relates to the nomenclature of Weber et al. [40] and the rs numbers relate to dbSNP (http://www.ncbi.nlm.nih.gov/snp/).

This set of 40 indels was previously validated as useful in ancestry estimation through the study of the HGDP-CEPH Diversity Panel [12], which is composed of 1,064 individuals from 52 different worldwide populations distributed in seven geographical regions [11]. The individual results have been deposited in the CEPH Genotype Database (http://www.cephb.fr/en/hgdp/main.php), from which they are available. The multiplex PCR assays and analysis in a MegaBACE 1000 DNA sequencer (GE Healthcare) of the indels were performed exactly as described previously [11].

### Data analysis

To estimate the proportion of Amerindian, European and African ancestry in each Brazilian, we applied a model-based clustering algorithm using the *Structure* software version 2.1 [41]. This software uses multilocal genotypes to infer the structure of each population and to allocate probabilistically the proportion of genomic ancestry of individuals in different populations. As parameters we assumed the admixture model, correlated allele frequencies and used 100,000 burn-in steps followed by 900,000 Markov Chain Monte Carlo iterations. We used for reference populations, 158 Europeans, 125 Sub-Saharan Africans and 107 Amerindians of the HGDP-CEPH Diversity Panel, which had been typed as part of our previous studies with the same set of 40 indels [8], [11].

Triangular graphs of the genomic proportions of Amerindian, European and African ancestry of each individual were obtained using the Tri-Plot program [42].

For statistical testing of the proportions of European, African and Amerindian ancestry in the different samples we developed a Monte Carlo resampling method, which has the advantage of being completely non-parametric [43], as follows. Since for each individual the relative proportions of European, African and Amerindian ancestries, must necessarily add to unity, a graph with a Cartesian coordinate system, in which the proportion of African ancestry (y-axis) is plotted against the proportion of European ancestry (x-axis) has the same information content as the triangular graph. We can now obtain in a two-dimensional Cartesian graph a point (P_i_), whose coordinate values are the mean proportion of European ancestry (X_i_; x-axis) and the mean proportion of African ancestry (Y_i_; y-axis) for sample i. To compare two samples (say, S_1_ and S_2_) we can use as a metric the distance between P_1_ and P_2_ in the two-dimensional graph space, which can be easily calculated, using the Pythagoras' theorem, as D  =  [(X1-X2)^2^ + (Y1-Y2) ^2^]^½^, i.e. the Euclidian distance between the means of the two samples. The probability distribution of the distance (D) was established empirically by randomization of the proportion of European ancestry of the two samples and also the proportion of African ancestry of the two samples, respectively, using the software Resampling Stats for Excel 3.2 (Resampling Stats, Inc., Arlington, Va., USA). The probability of random occurrence of a given value of the distance D was then established after 10,000 cycles. The significance level (two-tailed) was *a priori* established at α = 0.05.

## Supporting Information

Table S1 **Frequencies of the short allele of the 40 short insertion-deletion polymorphic loci used, in four regions of Brazil.**
(DOC)

Table S2 **To estimate the significance of the pairwise differences observed between the samples of the diverse regions we used a specially designed Monte Carlo randomization test of the Euclidian distance D between the means of the European and African ancestries.** In the table, the observed distances are in the cells above the diagonal and the probability of obtaining the observed distances by chance is shown in the cells below the diagonal. The cells in bold italic type are significant, even after Bonferroni's correction (P<0.008).
(DOC)
