# Supplementary material for: The Genomic Ancestry of Individuals from Different Geographical Regions of Brazil Is More Uniform Than Expected
Source: PLoS One. 2011 Feb 16;6(2):e17063. doi: 10.1371/journal.pone.0017063 (PMC3040205; doi:10.1371/journal.pone.0017063)
Supplement: Table S1 — Frequencies of the short allele of the 40 short insertion-deletion polymorphic loci used, in four regions of Brazil. (DOC) [file pone.0017063.s001.doc]

| **Region of Brazil** | | **North** | | | **Northeast** | | | **Southeast** | | | **South** | | |
| --- | --- | --- | --- | --- | --- | --- | --- | --- | --- | --- | --- | --- | --- |
| **Indels** | |  |  |  |  |  |  |  |  |  |  |  |  |
| MID# | dbSNP | **Eur.** | **Afr.** | **Amer.** | **Eur.** | **Afr.** | **Amer.** | **Eur.** | **Afr.** | **Amer.** | **Eur.** | **Afr.** | **Amer.** |
| **1** | **rs3917** | 0.367 | 0.250 | 0.182 | 0.358 | 0.338 | 0.162 | 0.310 | 0.234 | 0.247 | 0.329 | 0.205 | 0.135 |
| **15** | **rs4181** | 0.447 | 0.386 | 0.319 | 0.553 | 0.472 | 0.545 | 0.468 | 0.455 | 0.411 | 0.352 | 0.410 | 0.442 |
| **17** | **rs4183** | 0.500 | 0.531 | 0.556 | 0.590 | 0.683 | 0.596 | 0.557 | 0.474 | 0.479 | 0.341 | 0.667 | 0.584 |
| **51** | **rs16343** | 0.379 | 0.237 | 0.206 | 0.444 | 0.465 | 0.449 | 0.405 | 0.396 | 0.363 | 0.451 | 0.340 | 0.276 |
| **89** | **rs16381** | 0.513 | 0.601 | 0.696 | 0.583 | 0.616 | 0.643 | 0.570 | 0.584 | 0.527 | 0.591 | 0.653 | 0.673 |
| **107** | **rs16394** | 0.333 | 0.228 | 0.182 | 0.426 | 0.559 | 0.280 | 0.297 | 0.422 | 0.260 | 0.427 | 0.250 | 0.186 |
| **131** | **rs16415** | 0.457 | 0.461 | 0.332 | 0.580 | 0.616 | 0.522 | 0.538 | 0.545 | 0.384 | 0.628 | 0.435 | 0.359 |
| **132** | **rs16416** | 0.600 | 0.566 | 0.350 | 0.500 | 0.522 | 0.500 | 0.525 | 0.422 | 0.493 | 0.585 | 0.385 | 0.365 |
| **150** | **rs16430** | 0.333 | 0.430 | 0.453 | 0.371 | 0.493 | 0.623 | 0.348 | 0.390 | 0.472 | 0.352 | 0.378 | 0.436 |
| **159** | **rs16438** | 0.623 | 0.601 | 0.651 | 0.678 | 0.677 | 0.703 | 0.658 | 0.630 | 0.664 | 0.652 | 0.686 | 0.760 |
| **170** | **rs16448** | 0.453 | 0.443 | 0.280 | 0.445 | 0.404 | 0.343 | 0.437 | 0.461 | 0.397 | 0.610 | 0.288 | 0.256 |
| **258** | **rs16695** | 0.407 | 0.469 | 0.425 | 0.385 | 0.449 | 0.477 | 0.500 | 0.403 | 0.514 | 0.427 | 0.397 | 0.487 |
| **278** | **rs16715** | 0.657 | 0.592 | 0.514 | 0.595 | 0.608 | 0.419 | 0.582 | 0.571 | 0.466 | 0.707 | 0.442 | 0.403 |
| **420** | **rs140709** | 0.767 | 0.754 | 0.724 | 0.486 | 0.357 | 0.372 | 0.310 | 0.318 | 0.281 | 0.390 | 0.256 | 0.269 |
| **444** | **rs140733** | 0.228 | 0.386 | 0.491 | 0.361 | 0.400 | 0.481 | 0.323 | 0.377 | 0.453 | 0.256 | 0.433 | 0.494 |
| **468** | **rs140757** | 0.543 | 0.421 | 0.386 | 0.520 | 0.419 | 0.410 | 0.411 | 0.481 | 0.466 | 0.512 | 0.385 | 0.385 |
| **470** | **rs140759** | 0.327 | 0.289 | 0.243 | 0.372 | 0.356 | 0.265 | 0.278 | 0.318 | 0.308 | 0.329 | 0.314 | 0.237 |
| **663** | **rs1305047** | 0.490 | 0.561 | 0.617 | 0.473 | 0.500 | 0.530 | 0.525 | 0.448 | 0.562 | 0.524 | 0.487 | 0.583 |
| **788** | **rs1610874** | 0.893 | 0.798 | 0.762 | 0.467 | 0.368 | 0.404 | 0.494 | 0.539 | 0.459 | 0.519 | 0.427 | 0.468 |
| **857** | **rs1610942** | 0.723 | 0.588 | 0.552 | 0.647 | 0.601 | 0.622 | 0.696 | 0.643 | 0.568 | 0.704 | 0.532 | 0.487 |
| **914** | **rs1610997** | 0.300 | 0.311 | 0.300 | 0.620 | 0.595 | 0.609 | 0.506 | 0.396 | 0.445 | 0.500 | 0.423 | 0.340 |
| **918** | **rs1611001** | 0.403 | 0.417 | 0.346 | 0.569 | 0.486 | 0.558 | 0.399 | 0.474 | 0.479 | 0.451 | 0.360 | 0.327 |
| **1002** | **rs1611084** | 0.510 | 0.465 | 0.458 | 0.622 | 0.646 | 0.574 | 0.532 | 0.552 | 0.459 | 0.530 | 0.519 | 0.468 |
| **1054** | **rs2067180** | 0.557 | 0.474 | 0.401 | 0.412 | 0.424 | 0.405 | 0.513 | 0.513 | 0.466 | 0.506 | 0.365 | 0.325 |
| **1092** | **rs2067188** | 0.483 | 0.592 | 0.771 | 0.440 | 0.466 | 0.487 | 0.462 | 0.571 | 0.678 | 0.373 | 0.669 | 0.701 |
| **1100** | **rs2067217** | 0.280 | 0.325 | 0.327 | 0.191 | 0.221 | 0.169 | 0.285 | 0.299 | 0.281 | 0.311 | 0.288 | 0.321 |
| **1129** | **rs2067373** | 0.386 | 0.351 | 0.336 | 0.479 | 0.507 | 0.325 | 0.386 | 0.403 | 0.363 | 0.488 | 0.280 | 0.301 |
| **1291** | **rs2307548** | 0.490 | 0.434 | 0.425 | 0.431 | 0.303 | 0.333 | 0.449 | 0.364 | 0.404 | 0.494 | 0.393 | 0.410 |
| **1352** | **rs2307624** | 0.473 | 0.509 | 0.673 | 0.433 | 0.514 | 0.471 | 0.544 | 0.552 | 0.534 | 0.506 | 0.622 | 0.635 |
| **1428** | **rs2307733** | 0.437 | 0.338 | 0.241 | 0.385 | 0.329 | 0.250 | 0.291 | 0.409 | 0.236 | 0.427 | 0.301 | 0.214 |
| **1537** | **rs2307745** | 0.500 | 0.640 | 0.645 | 0.639 | 0.652 | 0.760 | 0.601 | 0.610 | 0.705 | 0.512 | 0.649 | 0.667 |
| **1549** | **rs2307782** | 0.377 | 0.272 | 0.212 | 0.418 | 0.500 | 0.351 | 0.297 | 0.364 | 0.219 | 0.482 | 0.269 | 0.217 |
| **1582** | **rs2307838** | 0.550 | 0.333 | 0.332 | 0.520 | 0.557 | 0.456 | 0.487 | 0.448 | 0.438 | 0.543 | 0.372 | 0.442 |
| **1642** | **rs2307850** | 0.280 | 0.298 | 0.405 | 0.253 | 0.331 | 0.327 | 0.310 | 0.390 | 0.397 | 0.293 | 0.404 | 0.372 |
| **1759** | **rs2307955** | 0.439 | 0.461 | 0.388 | 0.215 | 0.275 | 0.295 | 0.297 | 0.286 | 0.301 | 0.348 | 0.273 | 0.318 |
| **1763** | **rs2307959** | 0.466 | 0.548 | 0.584 | 0.417 | 0.355 | 0.519 | 0.386 | 0.513 | 0.507 | 0.488 | 0.560 | 0.519 |
| **1847** | **rs2308043** | 0.700 | 0.579 | 0.458 | 0.723 | 0.658 | 0.622 | 0.677 | 0.724 | 0.637 | 0.707 | 0.609 | 0.597 |
| **1861** | **rs2308057** | 0.860 | 0.355 | 0.448 | 0.230 | 0.158 | 0.250 | 0.190 | 0.299 | 0.315 | 0.268 | 0.314 | 0.351 |
| **1943** | **rs2308135** | 0.500 | 0.579 | 0.712 | 0.486 | 0.430 | 0.608 | 0.532 | 0.500 | 0.671 | 0.476 | 0.622 | 0.714 |
| **1952** | **rs2308144** | 0.303 | 0.276 | 0.269 | 0.392 | 0.468 | 0.426 | 0.310 | 0.364 | 0.288 | 0.360 | 0.359 | 0.260 |

Eur. = European

Afr. = African

Amer. = Amerindian
