# Supplementary material for: The Genomic Ancestry of Individuals from Different Geographical Regions of Brazil Is More Uniform Than Expected
Source: PLoS One. 2011 Feb 16;6(2):e17063. doi: 10.1371/journal.pone.0017063 (PMC3040205; doi:10.1371/journal.pone.0017063)
Supplement: Table S2 — To estimate the significance of the pairwise differences observed between the samples of the diverse regions we used a specially designed Monte Carlo randomization test of the Euclidian distance D between the means of the European and African ancestries. In the table, the observed distances are in the cells above the diagonal and the probability of obtaining the observed distances by chance is shown in the cells below the diagonal. The cells in bold italic type are significant, even after Bonferroni's correction (P<0.008). (DOC) [file pone.0017063.s002.doc]

|  | **North** | **Northeast** | **Southeast** | **South** |
| --- | --- | --- | --- | --- |
|  | **(Pará)** | **(Ceará)** | **(Rio de Janeiro)** | **(Rio Grande do Sul)** |
| **North** |  | 0.04845 | 0.13251 | 0.38021 |
| **(Pará)** |  |
| **Northeast** | 0.2528 |  | 0.10111 | 0.36227 |
| **(Ceará)** |  |
| **Southeast** | ***0.0001*** | 0.0126 |  | 0.26441 |
| **(Rio de Janeiro)** |  |
| **South** | ***0.0001*** | ***0.0001*** | ***0.0001*** |  |
| **(Rio Grande do Sul)** |  |
